# HF Etiology and cardiac contractility modulation therapy

**DOI:** 10.1186/s12872-024-03950-8

**Published:** 2024-05-29

**Authors:** Karapet Davtyan, Ivan Chugunov, Arpi Topchyan, Yury Mareev, Natalia Mironova, Elena Rimskaya, Sergey Golitsyn, Evgeny Mikhaylov, Dmitry Lebedev, Marianna Vander, Elena Lyasnikova, Maria Sitnikova, Khatuna Minjia, Svetlana Glembo, Oleg Sukhorukov

**Affiliations:** 1 National Medical Research Center for Therapy and Preventive Medicine, Moscow, Russia; 2 National Medical Research Centre of Cardiology after E.I. Chazov, Moscow, Russia; 3 Almazov National Medical Research Centre, Saint-Petersburg, Russia; 4 Research and Practical Center of Interventional Cardioangiology of Sechenov University, Moscow, Russia

**Keywords:** Heart failure, Optimizer, Device-based therapy, Heart failure etiology

## Abstract

**Objectives:**

Our study aimed to assess the safety and efficacy of cardiac contractility modulation (CCM) therapy in patients with heart failure with reduced ejection fraction (HFrEF) depending on HF etiology.

**Methods:**

We enrolled 166 patients with optimal medical therapy-resistant HFrEF (median age 59 years, 83.7% males, median NYHA class − 2, median left ventricular ejection fraction (LVEF) − 29.0%) who underwent CCM therapy device implantation from 2013 to 2019 in four medical centers in Russia. The HF etiology was determined based on invasive coronary angiography or cardiac MRI data. Transthoracic echocardiography (TTE), 6-minute walking test (6MWT), and NTproBNP-tests were performed at a baseline and 12 months after the implantation.

**Results:**

The ischemic etiology of HF was revealed in 100 patients (61.5%) (ICM group); the non-ischemic group (NICM) evolved 66 patients (38.5%). Patients in the ICM group were significantly older (61[57–69] vs. 55 [42.8–61], *p* < 0.001), more frequently had hypertension (79% vs. 42.4%, *p* < 0.001) and chronic kidney disease (43% vs. 22.7%, *p* = 0.012). Patients in the NICM group had significantly more often atrial fibrillation (AF) (58% vs. 74%, *p* = 0.048), larger end-diastolic volume (EDV) (249 [208–309] vs. 220 [192–271], *p* = 0.019) and end-systolic volume (ESV) (183 [147–230] vs. 154 [128–199], *p* = 0.003). There were no significant differences in mortality between ICM and NICM groups (14.4 vs. 10.8%, *p* = 0.51). In 12 months, there was a significant increase in LVEF in the NICM group (+ 2.0 [2–6] vs. +7.7 [2–12], *p* < 0.001), while the improvement in the 6MWT (+ 75 [22–108] vs. +80 [10–160], *p* = 0.851) and NYHA class did not reach the level of significance. The subanalysis between patients with improved NYHA class and those without improvement revealed that patients without improvement more frequently had AF (56% vs. 89%; *p* < 0.01), chronic obstructive lung disease (18% vs. 35% *p* = 0.047), higher blood pressure (110 [105–120] vs. 120[110–129]; *p* = 0.032).

**Conclusion:**

In this multicenter retrospective study, patients with non-ischemic HFrEF showed a significantly higher improvement in LVEF and LV reverse remodeling following CCM therapy device implantation. There was no significant association between HF etiology and survival in drug-resistant HFrEF patients following CCM therapy.

## Introduction

Cardiac contractility modulation (CCM) is a relatively new device-based (the Optimizer™ system by Impulse Dynamics, Orangeburg, NY, USA) therapy for heart failure with reduced ejection fraction (HFrEF). It can be recommended for patients with optimal medical therapy (OMT)-persistent symptoms of HF. CCM involves the administration of a biphasic non-excitatory electrical impulse to the interventricular septum during the ventricular absolute refractory period. CCM affects myocardial cell biology: in vitro studies showed that it enhances myocardial contraction via calcium circulation regulation without increasing myocardial oxygen consumption [1]. The results of clinical studies supported these data: the administration of CCM was associated with clinical improvement and lower hospitalization rates in patients with HFrEF (II-IV NYHA class, left ventricular ejection fraction (LVEF) < 40%) [2–6]. The meta-analysis of randomized controlled trials conducted by Giallauria et al. [7] revealed a moderate effect on peak oxygen consumption (VO_2_ peak) and exercise tolerance, thus improving the quality of life. Still, it demonstrated no effect on LVEF and mortality.

The impact of HF etiology on CCM therapy efficacy remains controversial. The etiology of heart failure (nonischemic vs. ischemic) was not associated with improvement in the etiology-dependent subgroup analysis of the FIX-HF-5 study [8]. On the contrary, the single-center study conducted by Fastner et al. reported a significant increase in LVEF and LV reverse modeling in NICM patients in the mid-term perspective [9]. A year later, Fastner et al [10] presented their findings in a larger cohort of patients. The authors did not reveal any significant difference in 12-month LVEF between ICM and NICM patients. These changes achieved the level of significance only in patients completing the 5-year follow-up.

Here, we present the 12-month results of our multicenter HF etiology-specified analysis of CCM therapy efficacy and safety in real-world practice.

## Methods

This multicenter retrospective analysis included data from 166 patients with OMT-resistant HFrEF and NYHA class II-IV who underwent CCM therapy system implantation using Optimizer™ IV or Optimizer™ Smart (Impulse Dynamics, Orangeburg, NY, USA) from 2013 to 2019 in four medical centers in Russia within the framework of the national CCM therapy device implantation program. The federal program protocol was initially approved by the National Ethics Committee and, after that, by each centers’ Independent Ethics Committee. All patients signed a written informed consent before recruitment.

The inclusion criteria were age ≥ 18 years, OMT-resistant HFrEF, and NYHA class ≥ II. The exclusion criteria were patients on the heart transplant waiting list, a history of myocardial infarction, PCI, cardiac surgeries within three months, acute myocarditis, hypertrophic cardiomyopathy, reversible causes for HF, mechanical tricuspid valve, severe comorbidities (acute decompensation, injury, or failure in other organ systems).

The implantation procedures were performed under local anesthesia with lidocaine hydrochloride (10 mg/ml). An infraclavicular incision was made, and a pocket was created in the right subclavian region. The leads via the subclavian approach were advanced to the heart. Patients implanted with Optimizer™ IV got three screw-in leads - one atrial (Boston Scientific 7741 Ingevity IS-1 52 cm) and two ventricular leads (Boston Scientific 7742 Ingevity IS-1 59 cm, St Jude Tendril STS IS-1 59 cm); patients with Optimizer™ Smart - only two ventricular leads (Boston Scientific 7742 Ingevity IS-1 59 cm, St Jude Tendril STS IS-1 59 cm). The atrial lead was fixed into the right atrial appendage; the two ventricular leads were implanted in the right ventricular aspect of the interventricular septum. The tip-to-tip distance between ventricular leads was ≥ 2 cm.

### Follow-up

The clinical follow-up duration was 12 months, with follow-up visits at 2, 6, and 12 months. Devices’ interrogation (Optimizer™ and CRT-D) and data analysis were performed at each follow-up visit. Data regarding clinical events and healthcare utilization were also collected. We thoroughly screened the available medical records of all 166 patients. The following parameters were collected at baseline and 12 months after implantation: demographics, medical history, physical examination data, laboratory examination data, NYHA class, six-minute walking distance (6MWD), transthoracic echocardiography (TTE), NTproBNP levels. Since February 2020, the follow-up protocol has been changed due to the COVID-19 pandemic. In some patients, the 12-month follow-up data was acquired remotely.

### Statistical analysis

Continuous variables were presented as median (Me), interquartile range (IQR), mean (M), and standard deviation (SD) depending on the distribution. Categorical variables were presented as frequencies (percentages). Differences in the patients’ continuous data results were checked for significance with the Wilcoxon signed-rank test or the Student’s T-test, respectively. The chi-square test was used to compare categorical variables. The correlation between metrics was evaluated by calculating the Spearman correlation coefficient. For survival analyses, Kaplan-Maier curves were done. A two-tailed p-value ≤ 0.05 was regarded to be significant. Data were analyzed with the R programming language.

## Results

### Baseline characteristics

Data from 166 patients with OMT-resistant HFrEF who underwent Optimizer device implantation from 2013 to 2019 in four medical centers in Russia were analyzed in this study. The median age of the total study participants was 59 [54.0;66.0] years; the majority were males (83.7%). The median LVEF was 29% [24.1–33.0], with a median NYHA class of 2 [2;3]. The ischemic etiology of HF was revealed in 100 patients (61.5%) (ICM group); the non-ischemic group (NICM) evolved 66 patients (38.5%). Patients in the ICM group were significantly older (61[57–69] vs. 55 [42.8–61], *p* < 0.001), more frequently had hypertension (79% vs. 42.4%, *p* < 0.001), and chronic kidney disease (43% vs. 22.7%, *p* = 0.012). Patients in the NICM group had significantly more often atrial fibrillation (AF) (58% vs. 74%, *p* = 0.048), larger enddiastolic volume (EDV) (249 [208;309] vs. 220 [192;271], *p* = 0.019) and endsystolic volume (ESV) (183 [147;230] vs. 154 [128;199], *p* = 0.003). They also more frequently received angiotensin receptor/neprilysin inhibitors [28.8% vs. 9%, *p* = 0.002] and anticoagulants [77.3% vs. 60%, *p* = 0.032]. Only a third of the patients (29.5%) were previously implanted with ICD/CRT-D due to the limited quantity of state-funded available devices. Baseline characteristics of patients in detail, as well as the number of available records for each parameter, are presented in Table 1.

**Table 1:** Baseline clinical, echocardiography characteristics and NTproBNP

	*n* = 166	ICM (*n* = 100)	NICM (*n* = 66)	*p*-value	*N*
Male, N (%)	139 (83.7%)	87 (87.0%)	52 (78.8%)	0.235	166
Age, Me (Q25; Q75)	59.0 [54.0;66.0]	61.0 [57.0;69.0]	55.0 [42.8;61.0]	< 0.001	166
BMI, Me (Q25; Q75)	29.0 [26.3;32.0]	29.0 [26.2;32.0]	29.0[26.8;31.8]	0.800	165
Hypertension, N (%)	107 (64.5%)	79 (79.0%)	28 (42.4%)	< 0.001	166
Diabetes, N (%)	38 (22.9%)	25 (25.0%)	13 (19.7%)	0.544	166
Chronic kidney disease, N (%)	58 (34.9%)	43 (43.0%)	15 (22.7%)	0.012	166
AF N (%)	107 (64,5%)	58 (58%)	49 (74%)	0.048	166
AF type N (%):					
Paroxysmal	56 (52.3%)	29 (50.0%)	27 (55.1%)		
Persistent	3 (2.80%)	1 (1.72%)	2 (4.08%)		
Permanent	48 (44.9%)	28 (48.3%)	20 (40.8%)		
COPD, N (%)	35 (21.9%)	22 (22.4%)	13 (21.0%)	0.980	160
Preexisting ICD, N (%)	39 (23.5%)	26 (26.0%)	13 (19.7%)	0.453	166
ICD at 12-month, N (%)	52 (31.3%)	35 (35.0%)	17 (25.8%)	0.278	166
Preexisting CRT, N (%)	10 (6.02%)	4 (4.00%)	6 (9.09%)	0.207	166
СRТ at 12 month, N (%)	11 (6.63%)	5 (5.00%)	6 (9.09%)	0.348	166
Systolic BP, Me (Q25; Q75)	110 [105;120]	120 [110;125]	110 [100;116]	< 0.001	165
Heart rate, Me (Q25; Q75)	73.0 [66.0;82.0]	72.0 [64.5;81.0]	75.0 [68.2;81.5]	0.245	165
Shortness of breath, N (%)	109 (99.1%)	60 (100%)	49 (98.0%)	0.437	110
Oedema, N (%)	48 (29.1%)	33 (33.3%)	15 (22.7%)	0.195	165
NYHA, Me (Q25; Q75)	2.00 [2.00;3.00]	2.00 [2.00;3.00]	3.00 [2.00;3.00]	0.088	166
6MWD, Me (Q25; Q75)	350 [300;400]	350 [300;400]	340 [300;390]	0.654	145
LV EDD, Me (Q25; Q75)	69.0 [64.0;73.0]	67.0 [63.0;72.0]	71.0 [66.0;76.0]	0.002	162
LV ESD, Me (Q25; Q75)	58.0 [52.0;64.0]	56.0 [51.0;61.2]	61.5 [57.0;66.2]	< 0.001	156
LV EDV, Me (Q25; Q75)	231 [196;285]	220 [192;271]	249 [208;309]	0.019	157
LV ESV, Me (Q25; Q75)	163 [134;210]	154 [128;199]	183 [147;230]	0.003	157
LVEF, Mean (SD)	29.0 [24.1;33.0]	30.0 [25.0;33.0]	28.0 [24.0;31.8]	0.149	166
ARNI, N (%)	28 (16.9%)	9 (9.00%)	19 (28.8%)	0.002	166
ACEi, /ARB/ARNI, N (%)	150 (96.8%)	87 (96.7%)	63 (96.9%)	1.000	155
β-blockers, N (%)	158 (95.2%)	95 (95.0%)	63 (95.5%)	1.000	166
MRA, N (%)	157 (94.6%)	95 (95.0%)	62 (93.9%)	1.000	166
SGLT2, N (%)	4 (2.74%)	2 (2.44%)	2 (3.12%)	1.000	146
Triple therapy for HF, N (%)	135 (87.1%)	79 (87.8%)	56 (86.2%)	0.956	155
Digoxin, N (%)	41 (25.6%)	21 (22.1%)	20 (30.8%)	0.294	160
Loop diuretics, N (%)	151 (91.0%)	94 (94.0%)	57 (86.4%)	0.161	166
Amiodaron, N (%)	34 (22.8%)	17 (20.0%)	17 (26.6%)	0.455	149
Anticoagulant, N (%)	111 (66.9%)	60 (60.0%)	51 (77.3%)	0.032	166
AF and anticoagulant, N (%)	101 (94.4%)	53 (91.4%)	48 (98.0%)		
NTproBNP, Me (Q25; Q75)	1388 [832;3010]	1228 [809;2738]	1516 [950;3966]	0.199	160

ACEi - Angiotensin-converting enzyme inhibitors, AF – atrial fibrillation, ARB – angiotensin receptor blocker, ARNI - angiotensin receptor neprilysin inhibitor, β-blockers - beta-adrenergic blocker, BMI – body mass index, BP – blood pressure, CRT – cardiac resynchronization therapy, EDD - end-diastolic diameter, ICD – implantable cardioverter defibrillator, HF – heart failure, LV EF - left ventricular ejection fraction, LV EDD - left ventricular end-diastolic dimension, LV EDV - left ventricular end-diastolic volume, LV ESD - left ventricular end-systolic dimension, LV ESV - left ventricular end-systolic volume, MRA - mineralocorticoid receptor antagonists, NTproBNP - N-terminal (NT)-prohormone B-type natriuretic peptide, SGLT2 - sodium-glucose Cotransporter-2 Inhibitors, 6MWD − 6-minute walking distance

Successful device implantation was carried out in all patients. During the 12-month follow-up, five patients developed major pectoral muscle pacing, requiring device lead replacement in three patients. In two patients, we set the device parameters so that the CCM pacing was continued with one lead, and the other was used only for sensing.

### CCM device pacing rate

**Fig. 1:**
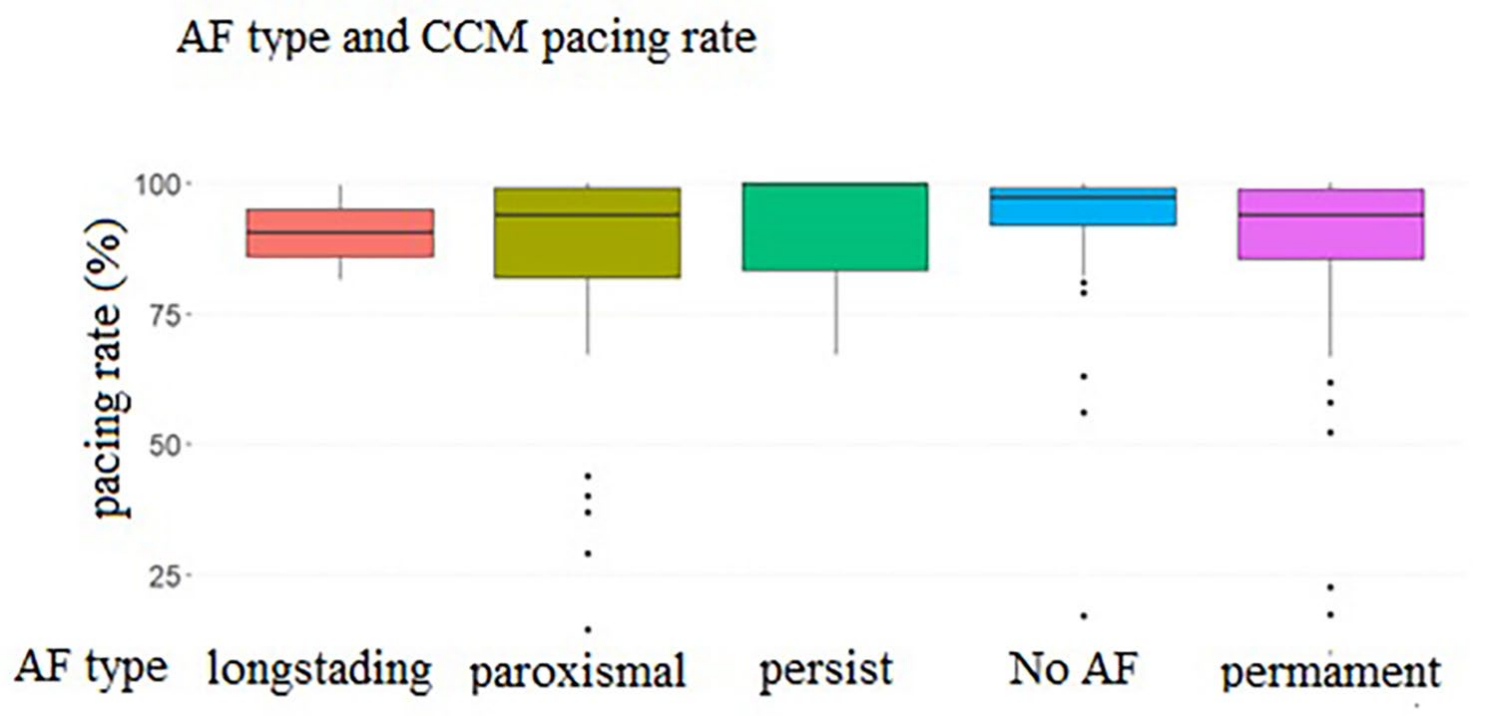
CCM pacing rates according to the rhythm

The CCM device pacing rate data was available for 134 patients (80.7%). The CCM pacing rate > 75% was achieved in 112 patients (83.6%); 86 of them (76.8%) have also reached the level of > 90% with a median rate of 95.2% [88.2;99.0]. The subanalysis of the CCM pacing rate in patients with AF showed a high Optimizer pacing rate regardless of AF type (Fig. 1).

### Survival

**Fig. 2:**
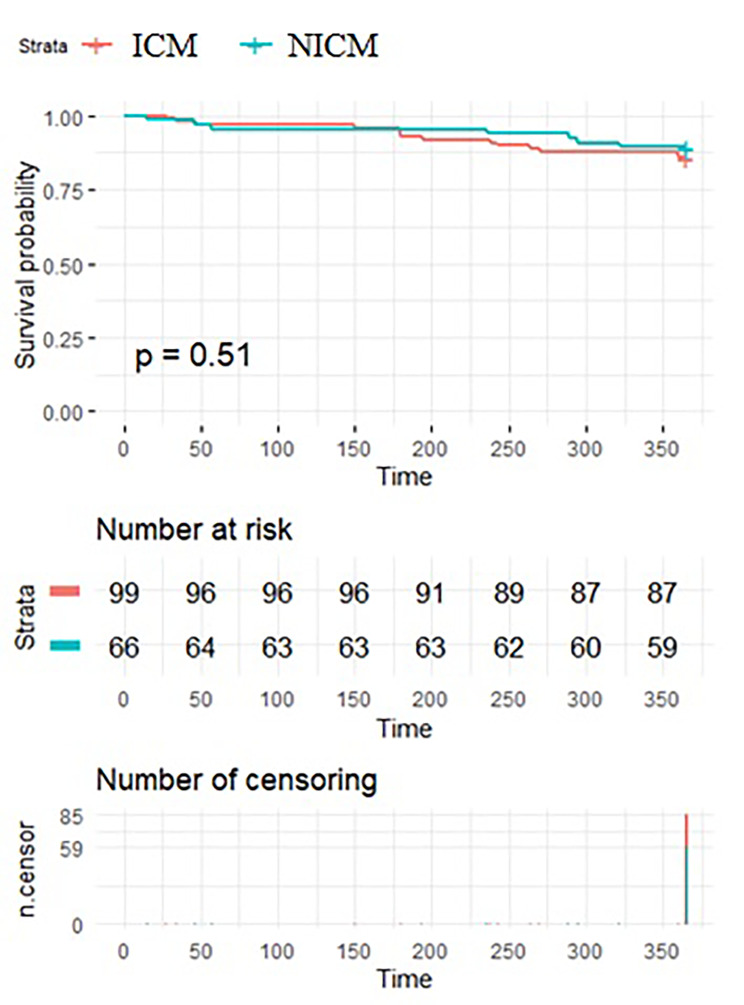
Survival of patients in ICM and NICM groups

21 patients (13%) died during the 12-month follow-up. Cardiovascular death was confirmed in 12 patients. 9 patients died due to acute decompensation of chronic heart failure; 3 patients developed sudden cardiac death (only one had a previously implanted ICD). No mortality differences existed between patients with ICM and NICM (14.4 and 10.8% *p* = 0.51) (Fig. 2).

### Changes in transthoracic echocardiography, six-minute walking distance, and NTproBNP parameters

The thorough analysis of changes in TTE parameters, 6MWD test, and NTproBNP levels is presented in Table 2. We missed 12-month follow-up data in some patients due to remote follow-up during the COVID-19 pandemic.

**Table 2:** Echocardiography parameters, NYHA, 6-minute walking distance, and NTproBNP in 12-month follow-up

Parameters (Me [Q25; Q75])	ICM	NICM	*p*-value	*N*_(ICM/NICM)_
LV EDD_12_,	65.0 [60.0;70.0]	66.0 [61.0;72.0]	0.356	134
LV ESD_12_	54.0 [49.0;61.0]	53.0 [49.0;63.0]	0.991	132
LV EDV_12_	224 [187;260]	220 [176;258]	0.460	135
LV ESV_12_	150 [119;187]	142 [114;178]	0.257	135
LVEF_12_	32.0 [27.0;37.0]	35.0 [30.2;41.8]	0.006	135
MR_12_	2.00 [2.00;2.00]	2.00 [2.00;2.00]	0.272	131
TR_12_	2.00 [1.00;2.00]	1.00 [1.00;2.00]	0.108	131
NTproBNP_12_	822 [311;1787]	678 [192;1340]	0.162	120
6MWD_12_	427 [375;480]	440 [350;535]	0.748	110
NYHA_12_	2.00 [2.00;2.00]	2.00 [2.00;2.00]	0.362	132
Δ NYHA	0.00[-1.00;0.00]	0.00[-1.00;0.00]	0.041	132 (81/51)
Δ 6MWD	75.0 [22.5;108]	80.0 [10.0;160]	0.851	96 (59/37)
Δ EDD	-2.00 [-3.00;1.00]	-1.00 [-8.00;1.00]	0.156	132
Δ ESD	0.00 [-4.00;1.75]	-5.00 [-13.00;0.00]	0.001	127
Δ EDV	-3.00 [-22.75;21.8]	-24.00[-69.50;6.00]	0.007	129
Δ ESV	-3.00 [-18.00;10.0]	-24.00[-74.50;0.00]	< 0.001	129
Δ LVEF	2.00 [-2.00;6.50]	7.75 [2.00;12.0]	< 0.001	135 (85/50)
Δ NTproBNP	-262.55 [-791.00;0.50]	-591.00 [-1532.00; -147.00]	0.091	117 (76/41)
Death, N (%)	14 (14.4%)	7 (10.8%)	0.659	166

LV EF - left ventricular ejection fraction, LV EDD - left ventricular end-diastolic dimension, LV EDV - left ventricular end-diastolic volume, LV ESD - left ventricular end-systolic dimension, LV ESV - left ventricular end-systolic volume, MRA - mineralocorticoid receptor antagonists, MR - mitral regurgitation, NTproBNP - N-terminal (NT)-prohormone B-type natriuretic peptide, SGLT2 - sodium-glucose Cotransporter-2 Inhibitors, TR – tricuspid regurgitation

**Fig. 3:**
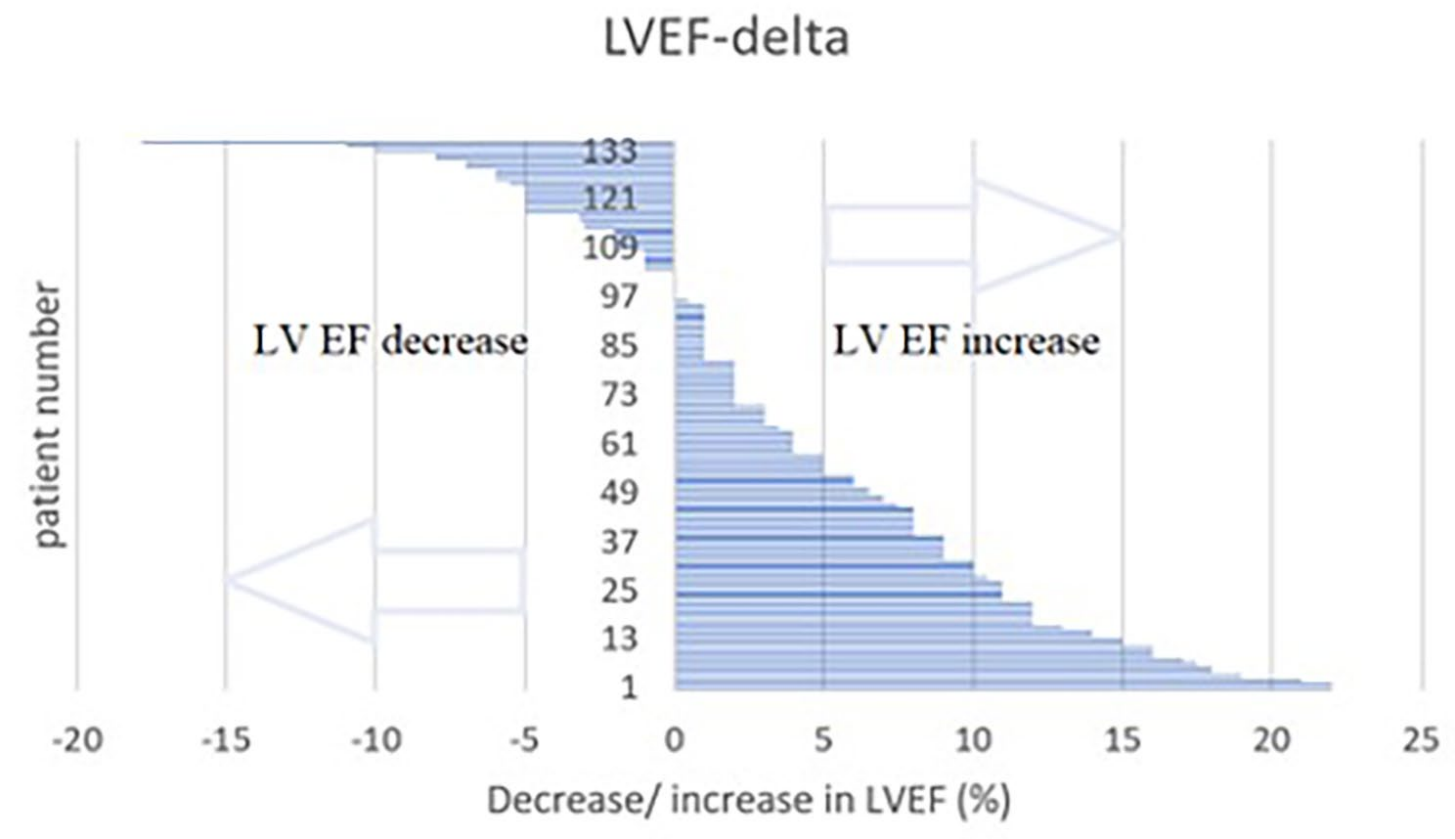
LVEF changes at 12-month follow-up

The overall 12-month LVEF was improved in most of the patients (Fig. 3). Moreover, 12-month LVEF in 16 patients (24%) in the NICM group increased up by ≥ 40%. The analysis of 12-month TTE data revealed a significant increase in LVEF (+ 2.0 [2.0;6.0] vs. +7.7[2.0;12.0], *p* < 0.001), and a significant decrease in end-systolic volume (-3.0 [-18.0;10.0] vs. -24.0[-74.5;0.0], *p* < 0.001) and end-diastolic volume (-3.0[-22.75;21.8] vs. -24[-69.5;6.0], *p* = 0.007) in the NICM group.

We detected an improvement in the 6MWT, but these changes did not reach the level of significance (+ 75 [22;108] vs. +80 [10;160], *p* = 0.851).

The NTproBNP level decreased in both groups, which was more pronounced in the NICM groups. However, this decrease did not reach statistical significance (-262.55 vs. -591.0, *p* = 0.091).

**Fig. 4:**
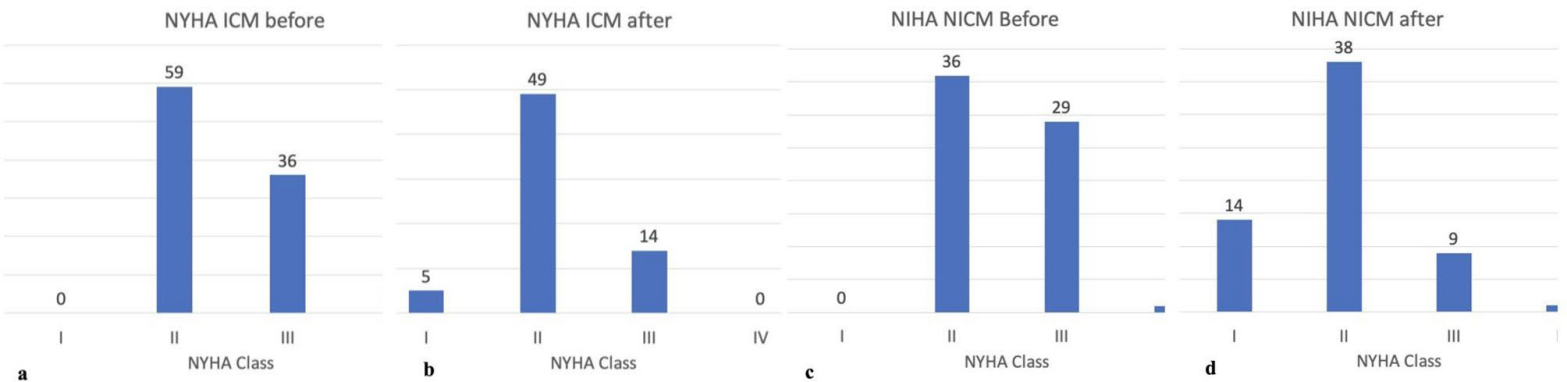
NYHA class at baseline and 12-month follow-up (**a**) baseline NYHA in ICM group, (**b**) 12-month NYHA in ICM group; (**c**) baseline NYHA in NICM group; (**d**) 12-month NYHA in NICM group

We revealed a modest NYHA class improvement in both groups, which was more significant in the NICM group (*p* = 0.041). Almost half of the patients completing 12 months of follow-up showed ≥ 1 NYHA class improvement (Fig. 4). Patients with improving/stable NYHA class significantly less frequently suffered from COPD (17.5% vs. 37%, *p* = 0.047), AF (56% vs. 89%, *p* = 0.010), and lower extremity edema (22.4% vs. 50%, *p* = 0.008). They also had lower baseline systolic blood pressure (110.0 [105.0;120.0] vs. 120.0[110.0;129.0], *p* = 0.032), baseline pulmonary artery systolic pressure (34.5[26.2;44.8] vs. 40.0[31.5;55.0], *p* = 0.030), ESD (57.0[52.0;63.0] vs. 61.0[56.0;68.0], *p* = 0.036), baseline NTproBNP (1279.0[665.0;2280.0] vs. 2472.0[1208.0; 6492.0, *p* = 0.001). The detailed comparative analysis of patients with improving/stable and worsening NYHA classes/fatal outcomes is presented in Table 3.

**Table 3:** Baseline clinical, echocardiography characteristics and NTproBNP in groups with and without clinical impairment

	*n* = 152	Patients without clinical impairment (*n* = 125)	Patients with clinical impairment (*n* = 27)	*p*-value	*n*
Age, Me (Q25; Q75)	59.0 [52.8;66.0]	59.0 [52.0;66.0]	61.0 [54.0;67.0]	0.383	152
Etiology, N (%):				0.726	152
ICM	94 (61.8%)	76 (60.8%)	18 (66.7%)		
NICM	58 (38.2%)	49 (39.2%)	9 (33.3%)		
Hypertension, N (%)	96 (63.2%)	78 (62.4%)	18 (66.7%)	0.844	152
Diabetes, N (%)	33 (21.7%)	30 (24.0%)	3 (11.1%)	0.224	152
CKD, N (%)	56 (36.8%)	45 (36.0%)	11 (40.7%)	0.808	152
AF, N (%):		69 (56%)	24 (89%)	0.010	
PAF	44 (29.7%)	33 (27.3%)	11 (40.7%)		
PersAF	3 (2.03%)	2 (1.65%)	1 (3.70%)		
Permanent	42 (28.4%)	30 (24.8%)	12 (44.4%)		
COPD, N (%)	31 (21.1%)	21 (17.5%)	10 (37.0%)	0.047	147
ICD baseline, N (%)	35 (23.0%)	26 (20.8%)	9 (33.3%)	0.250	152
ICD at 12 months, N (%)	48 (31.6%)	38 (30.4%)	10 (37.0%)	0.657	152
CRT baseline, N (%)	8 (5.26%)	6 (4.80%)	2 (7.41%)	0.627	152
CRT at 12 months, N (%)	9 (5.92%)	7 (5.60%)	2 (7.41%)	1.000	152
SBP, Me (Q25; Q75)	110 [105;120]	110 [105;120]	120 [110;129]	0.032	151
Oedema N (%)	41 (27.2%)	28 (22.4%)	13 (50.0%)	0.008	151
NYHA, Me (Q25; Q75)	2.00 [2.00;3.00]	2.00 [2.00;3.00]	3.00 [2.00;3.00]	0.123	152
6MWD, Mean (SD)	350 [300;400]	350 [300;400]	358 [300;390]	0.876	133
EDD, Me (Q25; Q75)	68.5 [64.0;73.0]	68.0 [64.0;73.0]	71.5 [65.0;77.8]	0.067	150
ESD, Me (Q25; Q75)	58.0 [52.0;64.0]	57.0 [52.0;63.0]	61.0 [56.0;68.0]	0.036	147
EDV, Mean (SD)	242 (62.1)	239 (60.6)	253 (68.5)	0.348	146
ESV, Me (Q25; Q75)	164 [134;210]	162 [134;207]	176 [137;214]	0.432	146
LVEF, Mean (SD)	28.5 (5.89)	28.4 (6.03)	28.7 (5.33)	0.816	152
SPAP, Me (Q25; Q75)	36.0 [28.0;45.0]	34.5 [26.2;44.8]	40.0 [31.5;55.0]	0.030	137
Mitral regurgitation, Me (Q25; Q75)	2.00 [2.00;2.00]	2.00 [2.00;2.00]	2.00 [2.00;3.00]	0.001	150
Tricuspid regurgitation, Me (Q25; Q75)	1.00 [1.00;2.00]	1.00 [1.00;2.00]	2.00 [1.25;3.00]	< 0.001	151
ARNI, N (%)	21 (13.8%)	17 (13.6%)	4 (14.8%)	1.000	152
ACEi/ARA/ARNI, N (%)	136 (94.4%)	112 (94.9%)	24 (92.3%)	0.631	144
B-blocker, N (%)	144 (94.7%)	119 (95.2%)	25 (92.6%)	0.627	152
MRA, N (%)	143 (94.1%)	116 (92.8%)	27 (100%)	0.218	152
Digoxin, N (%)	35 (24.0%)	27 (22.5%)	8 (30.8%)	0.521	146
Loop diuretics, N (%)	92 (94.8%)	71 (94.7%)	21 (95.5%)	1.000	97
**NTproBNP, Me (Q25; Q75)**	**1360 [809;2899]**	**1279 [665;2280]**	**2472 [1208;6492]**	**0.001**	**146**
Pacing rate at 6 months, Me (Q25; Q75)	96.0 [83.2;99.0]	96.0 [83.9;98.9]	98.0 [81.7;99.0]	0.615	128
Pacing rate at 12 months, Me (Q25; Q75)	96.0 [89.0;99.0]	95.4 [89.1;99.0]	98.5 [74.8;99.0]	0.855	123

ACEi - Angiotensin-converting enzyme inhibitors, AF – atrial fibrillation, ARB – angiotensin receptor blocker, ARNI - angiotensin receptor neprilysin inhibitor, β-blockers - beta-adrenergic blocker, BMI – body mass index, BP – blood pressure, CRT – cardiac resynchronization therapy, EDD - end-diastolic diameter, ICD – implantable cardioverter defibrillator, HF – heart failure, LV EF - left ventricular ejection fraction, LV EDD - left ventricular end-diastolic dimension, LV EDV - left ventricular end-diastolic volume, LV ESD - left ventricular end-systolic dimension, LV ESV - left ventricular end-systolic volume, MRA - mineralocorticoid receptor antagonists, NTproBNP - N-terminal (NT)-prohormone B-type natriuretic peptide, SBP - systolic blood pressure, SGLT2 - sodium-glucose Cotransporter-2 Inhibitors, 6MWD − 6-minute walking distance, PAF - paroxysmal atrial fibrillation, PersAF - persistent atrial fibrillation

## Discussion

The impact of HF etiology on CCM therapy is poorly studied and remains controversial. Such an important HF marker as LVEF was not assessed in RCTs as the primary endpoint of CCM therapy; most of the RCTs assessed exercise tolerance [2–5]. So, the interpretation of LVEF changes following Optimizer system implantation is understudied. Our analysis revealed that CCM therapy in NICM patients led to significant LVEF improvement with no statistically significant effect on 12-month survival. We also detected a significant improvement in the NICM group in other TTE data, such as EDV and ESV, reflecting the LV reverse remodeling. Our findings correlated well with the study results conducted by Fastner et al. [9], showing not only a remarkable LVEF improvement in NICM patients but also an LV reverse remodeling process reflected in LVEDD reduction.

On the one hand, these results could have been predictable. Although the SCD rates were similar for ICM and NICM patients [11], an older age, significantly more frequent comorbidities, worse response to HF drug therapy, a higher risk of cardiovascular death (especially due to myocardial infarction), and worse survival in patients with ischemic cardiomyopathy [12–15] suggest a poorer outcome of CCM therapy in those patients. However, Abraham et al. [8] did not reveal any association between HF etiology and patients’ improvement. LVEF improvement was not dependent on HF etiology in the study conducted by Müller et al., too [16]. On the other hand, the last study evolved patients with > 35% LVEF, which can be a possible explanation for such discrepancy in study results.

Our study also confirmed the tendency for the NTproBNP level to decrease, which was more pronounced in NICM patients. It is interesting to notice that NTproBNP level reduction tendency reached the level of statistical significance in the study conducted by Kuschyk et al. [17] evaluating the long-term efficacy (34.2 ± 28 months) of CCM therapy. This may reflect the long-term beneficial effects of CCM therapy on cardiac myocytes. However, Kuschyk et al. did not specify the HF etiology.

In our study, the 12-month 6MWD improvement did not reach the significance level. We also did not reveal any differences in 6MWD changes depending on HF etiology. Most of the studies, including RCTs, showed a significant increase in exercise tolerance, particularly in 6MWD [7, 18–22]. The involvement of more patients with advanced heart failure in our study can be the possible explanation for not reaching the significance level for 6MWD improvement during 12 months of follow-up. However, the 12 months of follow-up confirmed the more significant improvement of the NYHA in NICM patients.

Our subanalysis of patients’ characteristics with and without clinical impairment revealed, that patients without clinical improvement more frequently suffered from COPD, AF, and edema, and had higher baseline systolic blood pressure, baseline pulmonary artery systolic pressure, ESD, and baseline NTproBNP. Our results indicate the crucial role of patients’ thorough selection for effective CMM therapy, as patients with progressive congestive heart failure, edema, and frequent hospitalizations may not benefit from CCM therapy. Thus, we could recommend, that these patients should immediately be placed on the waiting list for heart transplantation skipping the CCM therapy device implantation. However, further larger, multicenter studies with a control group and optimal medical therapy are necessary to clarify these findings.

### Limitations

There are some possible limitations of this study: First, this is a retrospective analysis of the prospective collected data. These data were collected within the framework of a state-funded CCM therapy implementation program in Russia. The state-funded character of the program predefined the number of participants and the duration of the clinical follow-up visits. Second, optimal medical therapy has changed over the years; most patients were implanted with Optimizer devices before ARNI and SGLT2 inhibitors became available in Russia. Third, the Optimizer device provides information about the device pacing rate, which is not always the same as the CCM therapy rate. Fourth, due to the COVID-19 pandemic, some follow-up visits were done remotely, and we don’t have complete follow-up data for some patients.

## Conclusion

Patients with non-ischemic HFrEF showed a significantly higher improvement in LVEF and LV reverse remodeling following CCM therapy device implantation. There was no significant association between HF etiology and survival in drug-resistant HFrEF patients following CCM therapy.

## Data Availability

The data that support the findings of this study are available from the corresponding author upon reasonable request.
